# Exploring strategy differences between humans and monkeys with recurrent neural networks

**DOI:** 10.1371/journal.pcbi.1011618

**Published:** 2023-11-20

**Authors:** Ben Tsuda, Barry J. Richmond, Terrence J. Sejnowski

**Affiliations:** 1 Computational Neurobiology Laboratory, The Salk Institute for Biological Studies, La Jolla, California, United States of America; 2 Neurosciences Graduate Program, University of California San Diego, La Jolla, California, United States of America; 3 Medical Scientist Training Program, University of California San Diego, La Jolla, California, United States of America; 4 Section on Neural Coding and Computation, National Institute of Mental Health, Bethesda, Maryland, United States of America; 5 Institute for Neural Computation, University of California San Diego, La Jolla, California, United States of America; 6 Division of Biological Sciences, University of California San Diego, La Jolla, California, United States of America; Princeton University, UNITED STATES

## Abstract

Animal models are used to understand principles of human biology. Within cognitive neuroscience, non-human primates are considered the premier model for studying decision-making behaviors in which direct manipulation experiments are still possible. Some prominent studies have brought to light major discrepancies between monkey and human cognition, highlighting problems with unverified extrapolation from monkey to human. Here, we use a parallel model system—artificial neural networks (ANNs)—to investigate a well-established discrepancy identified between monkeys and humans with a working memory task, in which monkeys appear to use a recency-based strategy while humans use a target-selective strategy. We find that ANNs trained on the same task exhibit a progression of behavior from random behavior (untrained) to recency-like behavior (partially trained) and finally to selective behavior (further trained), suggesting monkeys and humans may occupy different points in the same overall learning progression. Surprisingly, what appears to be recency-like behavior in the ANN, is in fact an emergent non-recency-based property of the organization of the neural network’s state space during its development through training. We find that explicit encouragement of recency behavior during training has a dual effect, not only causing an accentuated recency-like behavior, but also speeding up the learning process altogether, resulting in an efficient shaping mechanism to achieve the optimal strategy. Our results suggest a new explanation for the discrepency observed between monkeys and humans and reveal that what can appear to be a recency-based strategy in some cases may not be recency at all.

## Author summary

How we think our brain solves a task and how it *actually* does often differ in surprising ways. In this work, we leverage artificial neural network (ANNs) models to study the development and evolution of problem-solving strategies on a working memory task. We show how behaviors arise in the ANNs and progressively evolve, first mimicking monkeys’ behaviors early in the learning process and then progressing to human-like behaviors. Analysis of the neural processing in ANNs reveals a surprising mechanism underlying the behaviors, different than the prevailing theory of how monkeys and humans solve the task and why their performance differs. We discover how repeated experiences mold network activity to generate specific behavioral patterns that account for the variations seen between humans and monkeys. These findings spur deep questions into how different species and artificial neural networks learn and store memory from repeated experience, and how this memory shapes network activity to generate subsequent behaviors.

## Introduction

Animal models are commonly used to help understand human biology [1], especially studies with non-human primates [2–6]. Non-human primate studies rely on the similarity between monkeys and humans, yet assumption of such similarities without validation can be problematic [7–11]. Wittig et al. brought to light one such case, revealing in a series of papers [8, 12] major problems with inferring human behaviors from findings in non-human primates. They showed that although humans and monkeys both proficiently solved a set of working memory tasks, closer examination of error patterns revealed significant discrepancies in problem solving strategies. In particular, for several tasks monkeys used what appeared to be a recency-based strategy while humans used a target-selective strategy. These results suggested an alternative strategy was used by monkeys, originating from differential use of language, understanding of the task rules, or working memory capacity and selectivity [8].

Artificial neural networks (ANNs) are another useful model system with particular advantages for understanding complex neural computations [13–22]. ANNs can learn to perform tasks and behaviors mirroring animals and humans and suggest fundamental principles of neural organization and dynamics that underlie these behaviors [13–15, 18, 20]. These insights are possible due to the ability of ANNs to capture important aspects of neural population dynamics while providing full access to all components of the network for analysis. Russo et al. [13] demonstrated an elegant example of this strategy, using ANNs to reveal how neural activity is precisely structured to avoid “tangling” or ambiguous neural states that would be problematic for smooth and predictable motor activity. Roach et al. [14] demonstrated the other major advantage of ANN models: the ability to vary network parameters flexibly and at scale to understand relationships between these parameters, network dynamics, and output behavior. Roach et al. varied the specificity motifs of inhibitory connections in ANNs and showed how this parameter affects speed and accuracy of decision making by the network by altering the underlying network dynamics. Recently, recurrent neural networks (RNNs) and particularly a subset of RNNs that incorporate gating mechanisms called long short-term memory networks (LSTMs), have been used to understand how networks of neurons can perform tasks requiring working memory and long-term memory mimicking the behaviors and neural dynamics of animals and humans [16, 18]. In this work, we utilize LSTMs to investigate how task learning and neural network capacity affect the ANN’s problem-solving behavior and uncover the neural activity structure underlying the ANN’s solution, suggesting a plausible explanation for both humans’ and monkeys’ behaviors.

ANNs have proven particularly useful in understanding how large populations of neurons represent information [13, 23–26]. Multiple studies have shown that the geometry of how neural populations represent information in high dimensional activity space—often referred to as representational geometry—can have a major impact on both functional outputs and the types of errors made [27, 28]. ANNs have been successfully employed to elucidate and characterize neural population representational geometries: Sorscher et al. discussed how transformations of representational geometry in the visual pathway enables few-shot learning, and proposed a theory of how geometric relationships of neural population responses support this feature and predict identification errors [24]; Tsuda et al. demonstrated how different modes of neuromodulation (chemical vs electrical) can transition network activity along different activity manifolds resulting in independent effects on network dynamics and output behavior [25]; Gallego et al. discussed how neural manifolds defined in neural activity space help explain motor planning activity and behavior, with reference to several examples in which ANN models were used to elucidate mechanisms underlying recorded neural population activity data [26]. Together, these studies and many others have shown how ANNs can be used to help understand distributed representations coded in large populations of neurons.

Using RNNs, we show that a plausible explanation for the observed differences in behavior between monkeys and humans may be based on developmental differences. We show that RNNs trained on a working memory task can exhibit human-like or monkey-like behaviors, and in fact progress through these behaviors with increasing experience. Analysis of the mechanisms underlying the RNNs’ behavioral progression reveals that what appears to be a recency-based strategy is not recency-related, but rather stems from the developmental progression of the geometry of the RNNs’ solution state space, which, depending on the RNN’s experience, can generate both recency-like or target-selective behavior. Finally, we examine the role of a true recency-based strategy on task learning and performance.

## Results

### Wittig et al. 2016 [8] working memory tasks, experiments, and results

To investigate how monkeys and humans solve tasks requiring working memory, Wittig et al. [8] designed three types of working memory tasks which were called “Same-Different,” “Match-First,” and “Match-Any.” Each task involved presentation of a sequence of visual cues. After each sequence the subject (monkey or human) was prompted to respond “yes” or “no” as to whether the sequence just shown was a “match” type trial or not. The criteria for being a “match” varied in each of three tasks as described below.

**Fig 1:**
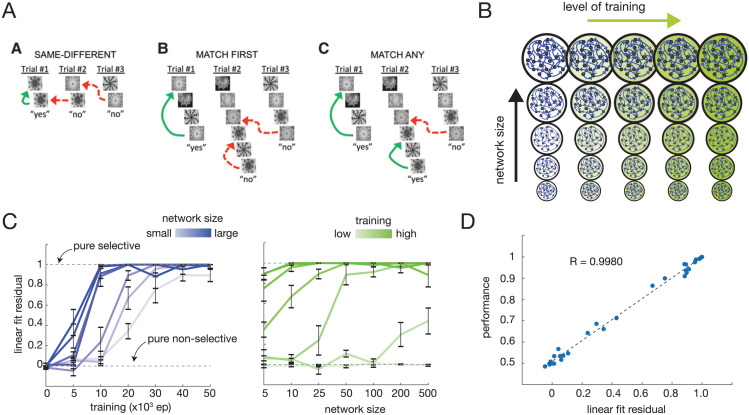
Task performance and target-selectivity increases as a function of both network size and training experience. (A) Working memory tasks from Wittig et al. 2016. In each trial a sequence of cues is presented and the subject must response “yes” or “no” as to whether a “match trial” occurred. For each task green arrows indicate correct match trials and dotted red lines indicate causes for erroneous “yes” responses in “no-match trials.” Reprinted from [8]. (B) Ashby chart schematic of modeling approach to characterize contribution of network size and training experience to task performance and strategy. (C) Linear fit residuals of serial position curves across network sizes and training experience, here shown for Same-Different (set size (i.e., possible cue types) 2). Linear fit residual mirrors performance progression, with larger networks’ linear fit residuals increasing more rapidly with increasing training experience. Error bars are SEM over 10 independently trained RNNs. (D) Linear fit residual and performance are tightly correlated across network configurations.

In Same-Different, each trial was a series of 2 cues (Fig 1A left). If these cues were the same, it was a “match” trial and a “yes” response was correct (indicated by leftmost green arrow of Same-Different examples in Fig 1A). If the cues were different, it was a “no-match” trial and a “no” response was correct. Mistakes could be made if the subject responded yes due to presentation of the same cue in a prior trial (red arrows in Same-Different examples in Fig 1A).

In Match-First, each trial was a series of 4 to 8 cues (Fig 1B middle shows example trials). If the first cue of the sequence was the same as the last cue, it was a “match” trial and a “yes” response was correct (indicated by green arrow of Match-First examples in Fig 1A). If these cues were different, it was a “no-match” trial and a “no” response was correct. Mistakes could be made if the subject responded “yes” due to presentation of the same cue in the middle of the sequence within a trial (referred to as “distractor cues”) or in a prior trial (red arrows in Match-First examples in Fig 1A).

In Match-Any, each trial was also a series of 4 to 8 cues (Fig 1A right). In this task, if the last cue presented in the trial matched any of the previous cues in that trial sequence it was a “match” trial and a “yes” response was correct (indicated by leftmost and middle green arrow of Match-Any examples in Fig 1A). If the no cues in the current sequence matched the last cue, it was a “no-match” trial and a “no” response was correct. Mistakes could be made if the subject responded yes due to presentation of the same cue in a prior trial (red arrow in Match-Any examples in Fig 1A).

For each task, the number of different possible cues used was varied (referred to in Wittig et al. 2016 as cue set size, e.g. 5 different cues vs 100 different cues; for clarity we also refer to this as “possible cue types”) and for Match-First and Match-Any the length of the trial could be varied between a sequence of 4 cues and a sequence of 8 cues (referred to trial size). For all trial types we refer to the last cue in a sequence as the “test cue” and the first cue in the sequence as the “lead cue” of the trial. “Target cue” refers to the cue(s) that when matching the “test cue” (last cue) would result in a match trial.

**Fig 2:**
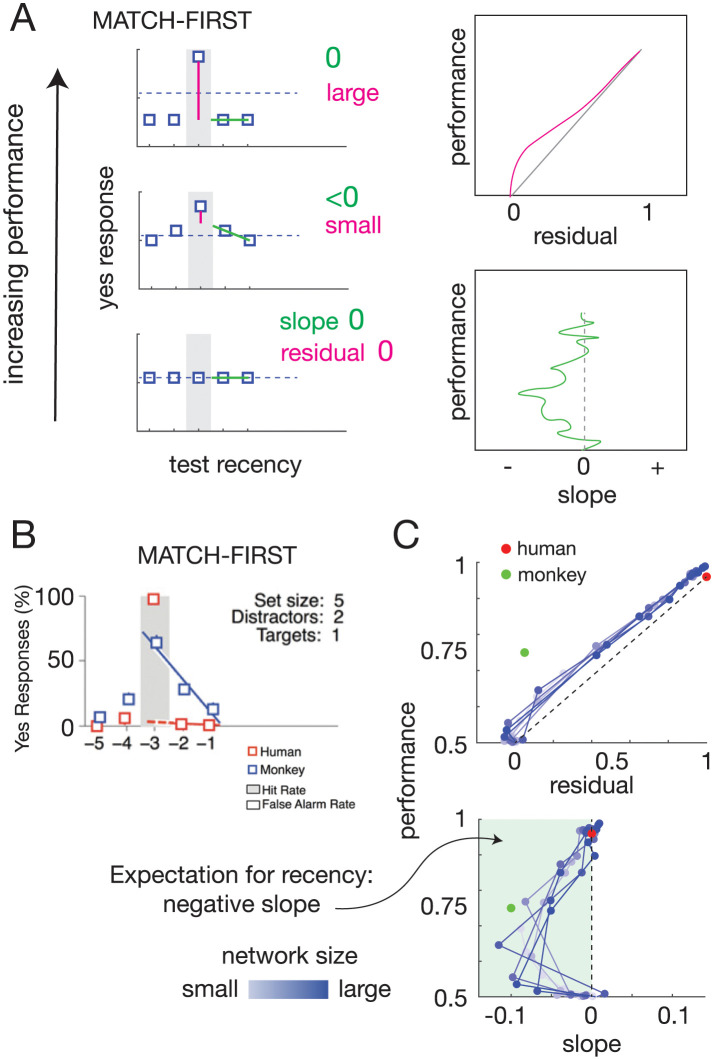
RNNs progress from random to recency-like to target-selective behavior over training. (A) Serial position curve exhibits specific characteristics depending on strategy. Left panels shows schematic of yes response rate across trial positions in a task (here, Match-First) with serial position curve (green line and text indicating magnitude of slope) and residual (magenta line and text indicating magnitude). Grey shaded position is the correct match position, i.e., the target cue. Random behavior would be characterized by a near-zero linear fit residual and slope; recency-based strategy would continue to have a small residual, but a slope of a particular direction—for Match-First, a negative slope; target-selective strategy would have a large residual and near-zero slope. Right panels, Progression through these strategies would be characterized by the residual and slope vs performance schematic plots. (B) Serial position curve with linear fits from Wittig et al. 2016 for Match-First with set size 5 (i.e., possible cue types) and trial size 4 (1 target cue, 2 distractor cues, 1 test cue). Serial position curve for monkey behavior shown in blue; for human behavior in red. Grey shaded position same as (A) indicates correct match position (target cue). Yes response rate at the target cue position is called “hit rate” in Wittig et al. 2016. Humans exhibit a target-selective behavior with large residual and near-zero slope. Monkeys exhibit a recency-based behavior with small residual and expected negative slope. Reprinted from [8]. (C) RNNs across the size spectrum (small to large is light to dark blue) exhibited a progression from random (zero residual, zero slope) to recency-like (small residual, negative slope) to target-selective behavior (high residual, zero slope). Each point represents average performance of 10 independently trained RNNs with the same parameter set. Points representing human and monkey residuals and slopes are shown in red and green, respectively.

To examine how monkey and humans were performing the tasks, Wittig et al. not only measured performance (which was similarly proficient between humans and monkeys for most of the task variations), but also specifically analyzed the types of errors made. To show this, they defined a “serial position curve” for each subject’s performance on a task. This curve was made by sorting trials based on how recently the test cue was last presented and measuring the “yes” response rate at each position. An example of a serial position curve for Match-First is shown in Fig 2B. In this case, when the test cue was previously presented in the immediately prior position (the -1 position on the x-axis), monkeys mistakenly responded yes at a rate indicated by the black box at the -1 position. Monkeys and humans had the highest yes response rate at the -3 position in this case, since these were correctly performed trials: responding “yes” when the test cue matched the first cue of the sequence (indicated by the grey shading in Fig 2B). To describe the trend in “yes” response rate by the position a cue was last presented, a linear fit was done using ≥2 points (blue line fit for monkeys and red dotted line fit for humans in Fig 2B).

As can be seen from the serial position curve shown in Fig 2B, monkeys and humans sometimes had different serial position curves indicating different types of errors made, despite often similar end performance. In particular, Wittig et al. revealed that monkeys often had an upward trending slope of their serial position curve proximal to the target cue position. For example in Fig 2B this can be seen as the negative slope from position -3 to position -1, with higher “yes” response rates closer to the target position (-3 position in this example). Wittig et al. observed this could represent a “recency-based strategy,” i.e., the likelihood of a yes-response was proportional to the proximity of the last match to the target cue position (the “yes” response rate is higher for position -2 than -1 in Fig 2B). In contrast, humans most often had a flat serial position curve, indicating the same likelihood of erroneous “yes” response across all non-correct positions: the near-zero slope of the red-dotted line in Fig 2B. This error rate profile indicated a “target-selective strategy,” since subjects exhibiting this error profile only had an increased “yes” response rate at the relevant position, i.e., the target cue position (e.g. red box in grey shaded position in Fig 2B). In addition to the slope of the serial position curve, the residual of the curve, calculated as the distance between the extrapolated linear fit and the observed yes-response rate at the target cue (grey shaded position) can also be used to indicate the degree of recency vs target-selective strategy being used. An example is shown in Fig 2A on the left where the serial position curve linear fit is shown with a green line and the residual is shown with a magenta line. More target-selective strategies (Fig 2A top left graph) result in large residuals whereas recency-based strategies result in smaller residuals (Fig 2A middle left graph).

### Modeling the contribution of capacity and task understanding

To investigate the contribution of capacity and task understanding on behavior and task performance, we modeled changes in capacity by varying network size and task understanding by varying training experience. We used the same working memory tasks introduced in Wittig et al. 2016 [8] (see Methods) to reveal behavioral differences between monkeys and humans (Fig 1A). ANNs allow systematic characterization of network behavior as a function of both network size and amount of experience, i.e. training. We varied RNNs (LSTMs in our implementation; see Methods for details) across a size spectrum (*n* ∈ {5, 10, 25, 50, 100, 200, 500} neural units) and training experience spectrum (*n*_*ep*_ ∈ {0, 5, 10, 20, 30, 40, 50} x 10^3^ trial episodes) (Fig 1B), which effectively captured the full range of performance on the task.

We found that for some tasks (e.g. Same-Different) networks across the size spectrum tested could gain proficiency, though different sized networks required different amounts of training. Across all tasks, smaller networks generally required more experience to gain comparable proficiency (S1 Fig). Following the metrics used by Wittig et al., we measured serial position curves [8] for networks with each size and experience combination. To assess the degree of target-selectivity of each network configuration, we measured the linear fit residual of each serial position curve, following the linear fit protocol of Wittig et al. (larger residual indicates higher target-selectivity; see Methods). We found that the linear fit residual increased more quickly with experience for larger networks, correlating very tightly with performance (Fig 1C and 1D and S2 Fig), indicating that higher target-selectivity was directly linked to higher performance.

### RNNs progress from random to recency to selective behavior with experience

Serial position curve linear fit residuals indicate the degree of target selectivity, but they cannot distinguish between different forms of non-selectivity. To illustrate this point, consider two scenarios: 1) a network begins untrained with random behavior, gradually increases selectivity with no other response trends, and finally achieves high target-selectivity (S3A Fig); 2) a network begins untrained, adopts a recency-like response profile, then achieves high target-selectivity (Fig 2A and S3B Fig). In both these cases, the linear fit residual is small while the network has a random behavior or a recency-based behavior and may follow very similar linear fit residual progressions. To distinguish between these possibilities, we introduced another measure of serial position curves: linear fit slope.

Linear fit slope distinguishes between the two scenarios above: in the first (random → low target-selective → high target-selective), linear fit slope will remain around zero (S3A Fig), whereas in the second (random → recency-based → high target-selective), linear fit slope will follow a predictable deflection during recency-based behavior (Fig 2A and S3B Fig). Importantly, the predicted recency-based behavior provides a strong prior on the direction of the slope deflection, decreasing the probability of erroneously attributing a spurious slope deflection to a strategy difference.

Using both measures—linear fit residual and linear fit slope—we assessed the networks on the working memory tasks. For some tasks, a clear progression emerged, following that predicted by the random → recency-based → high target-selectivity scenario (Fig 2C), while other tasks appeared to follow the alternative progression (random → low target-selective → high target-selective) (S4 Fig). Furthermore, in some cases networks across all sizes tested exhibited the recency-based progression (Fig 2C), while in others there appeared to be a size dependency, with smaller networks more likely to progress through recency-based behavior (S4 Fig). Three possibilities may underlie these discrepencies. First, different network configurations may truly follow different behavioral progressions. Second, there appeared to be a trend where task scenarios involving larger cue deck sizes (100 cues vs 5 cues) showed less recency-based progression. A simple reason for this may be related to sample size. Serial position curves rely on occurrences of “yes” responses at each position for each cue. We found evidence of such undersampling, revealing similar recency-based progression when sample size was increased proportional to cue deck size (S5A Fig). Third, in some cases networks—particularly larger networks—may progress through different strategies rapidly during training, such that if the sampling rate during training is too coarse, deflections of the residual or slope curves will be missed. We found evidence of this form of undersampling as well (S5B Fig).

**Fig 3:**
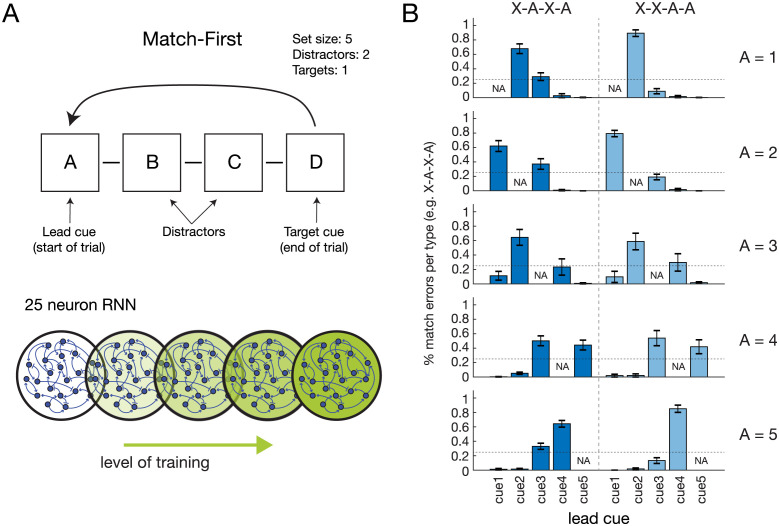
Stimulus-specific breakdown of match errors suggests not mere recency. (A) In a Match-First trial, a sequence of cues is presented and the agent must respond “yes” or “no” match by comparing the lead (cue A) and target (cue D) cues in the sequence. For a set size (i.e., possible cue types) of 5, there are 5 unique cues which we refer to as cue1 through cue5 (1–5, for short). We focus our analysis on the 25 neuron RNN which exhibited behavioral variation mimicking monkeys and humans over training on Match-First. (B) In the cue sequence labels, the number/letter represents the cue identity, A represents the cue of interest, and X represents any cue other than A. E.g. in the top row A = 1; dark blue bars indicate X-1-X-1 cue sequences (2–1-X-1, 3–1-X-1, 4–1-X-1, 5–1-X-1 from left to right) and light blue bars indicate X-X-1–1 cue sequences (2-X-1–1, 3-X-1–1, etc). A identities for each row is given on the right. For RNN trained on 20k trials and exhibiting recency behavior, both dark and light bars show uneven distribution of match errors across different lead cues. This refutes a simple recency-based strategy which would predict parity across all lead cues, i.e., 25% (horizontal dotted line in each graph) for all dark blue bars and 25% for all light blue bars. Error bars are SEM over 10 independently trained RNNs.

Although we had hypothesized that network size (capacity) and experience (learning) may contribute to strategy adoption and progression, it was nevertheless surprising that networks appeared to progress through a recency-based behavior given no recency-based reward scheme was used in the reinforcement learning structure of the tasks (see Methods). To investigate why the networks appeared to transition through a recency-based intermediate behavior, we performed in-depth analysis of the network behavioral progression from random to recency-based to high target-selectivity, focusing on the 25 neural unit RNN trained on the Match-First task with small cue deck size (5 cues) and 4-step trial size (2 distractors) (Fig 2B and 2C). In this task, trials take on the form A-B-C-D where a Match occurs when D and A are the same cue and no-match occurs otherwise (Fig 3A). Recency-based behavior was characterized by a higher X-A-X-A error “yes” response rate than X-X-A-A (where A is a given cue and X is any cue other than A; this relative difference causes the negative slope deflection in Fig 2C).

### Recency or something else?

At intermediate training experience, RNNs’ serial position curves exhibited the same residual and slope profiles characteristic of monkeys in Wittig et al. 2016. Our RNN models allowed systematic interrogation of the network behaviors, specifically the hypothesis that this behavior is reflective of a recency-based strategy, a hypothesis that has concrete predictions which can be validated or refuted in our model. Like monkeys, RNNs emergently exhibit this behavior, without an explicit recency-based reward scheme. A straightforward measure of whether a true recency-based strategy is being used is comparison of different cues sequences in which the lead cue is varied. A recency-based strategy not only predicts more X-A-X-A match errors than X-X-A-A in Match-First, but also predicts that all X-A-X-A errors are equally likely, regardless of the other cues’ (X) identities; 2-**1**-3-**1** and 5-**1**-3-**1** match responses should equally contribute to recency-based errors, where **1**-X-X-**1** is the correct match, and numbers indicate specific cues (i.e., “1” refers to cue 1).

To assess this, we measured the cue-specific match error responses of RNNs exhibiting “recency-strategy” behavior. A recency-based strategy would predict a flat rate of match error responses across all lead cue identities. For example, 2-1-X-1, 3-1-X-1, 4-1-X-1, and 5-1-X-1 would each make up 25% of X-1-X-1 match errors. Interestingly, this was not the case. Lead cue-specific responses varied strongly, ranging from 89% to 0%. This refutes a simple recency-based strategy. Furthermore, match error rates exhibited a ramping progression around the corresponding mistaken match cue (Fig 3), suggesting an underlying lead cue-specific structure to the match error rates. This was true in both the higher (X-A-X-A) and the lower (X-X-A-A) match error categories (Fig 3 dark and light blue bars, respectively). Although course-grain analysis suggested a recency-based strategy, the strong variation in response rate by lead cue identity reflects an underlying non-recency behavior derived from the formation and refinement of the RNNs’ solution state spaces.

### Formation of the RNN solution state space with experience

To understand the RNN’s solution state space, we first considered the population activity of the network over the course of individual trials. While clear differences could be seen in network activity trajectories over trials as training increased (compare 0k training episodes to 20k to 50k in S6 Fig), discerning relationships between RNN state space representations and behavior required analysis of RNN’s state space at each stage within a trial.

**Fig 4:**
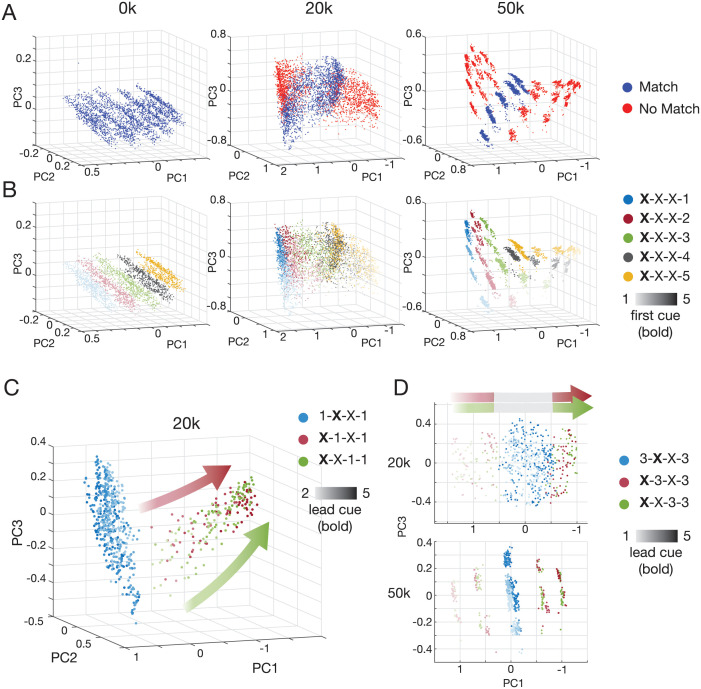
RNN state space geometry explains uneven match error profiles. (A) PCA of network activity after presentation of all cues in a trial for trials answered correctly, colored by match (blue) or no match (red) identity. The untrained RNN (0k) outputs random behavior (e.g. always “match”; so correct trials are the match trials). At 20k training episodes, distinct match and no match clusters begin to form. At 50k, clearly demarcated clusters in activity space are apparent, defining a “match” band (blue region) within a surrounding “no match” zone (red region). (B) Same plots as (A) but colored by last cue (target cue) and shaded by first cue in sequence. Color and shading shows clear organization at 50k; each cluster corresponds to a unique lead and last cue combination. Emergence of this conformation can begin to be seen at 20k. (C) PCA of state space for match error trials with target cue 1 shows arrangement that leads to mostly likely error overlaps with cue sequences with lower lead cues (lighter shaded red and green). (D) Neighboring match error trials for target cue 3 sequences shows similar result, with most likely overlap for cues starting with cue 2 or 4. For comparisons in (A),(B) PCA was performed on all correct trials (51% for 0k, 83% for 20k, 97% for 50k). For (C),(D) PCA was performed on all relevant trials (e.g. for (C), all 1-X-X-1, X-1-X-1, X-X-1-1 trials). Top 3 PCs explained >87% of activity variance for all analyses. In (D), PCs 1 and 3 explained 75% and 83% of variance for 20k and 50k trained RNNs, respectively.

To better understand the structure of the RNN’s solution state space, we considered the final activity state of the RNN after being presented with all four cues (first cue, 2 distractors, test cue). Using principal component (PC) analysis to analyze how network activity varied most (top 3 PCs accounted for >87% variance), we found a precise structure that emerges through training. Untrained RNNs adopt random behavior, for example always responding “match” leading to final state activity reflective only of stimulus-dependent effects (i.e., similarity-based influence on network state, Fig 4A and 4B, 0k). After some training when the recency-like behavior emerges (20k training episodes), activity states begin to show clustering reflective of “match” and “no match” regions (Fig 4A, 20k) suggesting some organization by target cue and first cue (Fig 4B, 20k). Finally, when target-selectivity is achieved (50k training episodes), easily distinguishable point clusters emerge representing a solution state space map underlying network behavior, with tightly defined activity spaces demarcating particular match and no match combinations (1-X-X-1 vs 2-X-X-1 vs 2-X-X-2, etc.) that follows a clear organization based on first and target cues (colored and shaded point clouds in Fig 4A and 4B, 50k). We found this topological map of RNNs’ activity state space was preserved across RNNs independently trained on the task (S7 Fig).

Further dissection of the RNN activity state space revealed that the arrangement of these network activity point clouds depends on cue-encoding, i.e., the final network state for 1-X-X-1 is closer to 2-1-X-1 than to 5-1-X-1 (blue, light red, and dark red points in Fig 4C, respectively), something that would not be predicted by a recency-based strategy. This also occurred for X-X-1–1 activity states (light vs dark green points in Fig 4C), as well as for the other target cues (Fig 4D shows for target cue 3). Less separated activity states after 20k training episodes resulted in more errors than after 50k training episodes, though the same structure remained (Fig 4D, 20k vs 50k). This closer activity state proximity (resulting in higher activity region overlap and consequent erroneous network readout) of particular cue sequences explains why the proportion of X-1-X-1 match errors was much higher for 2–1-X-1 (68%) than for 5–1-X-1 (0.3%) (Fig 3, dark blue bars, first row), and were not close to the 25% parity predicted by a recency-based strategy.

### Alignment of activity space sorting directions underlies recency-like phenotype

Although this insight into state space structure explained the lead cue-mediated inequality of match-error response profile, it did not help explain why networks consistently exhibited a recency-like response behavior. Given that final network state was organized by cues that were presented, we investigated how sequential cue presentation in a trial was encoded by the network. To do this, we considered the state of each network after being presented with the first 3 cues (lead cue and 2 distractor cues) and assessed whether network activity could be separated by cue history.

**Fig 5:**
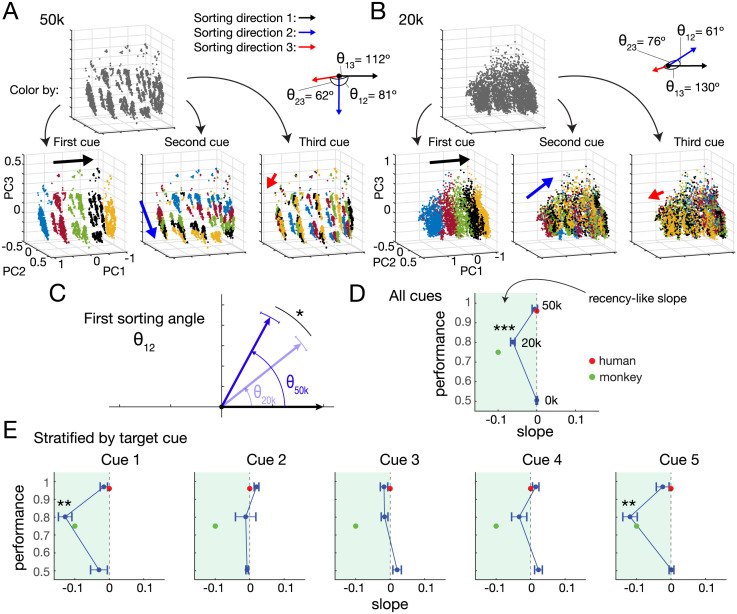
Cue-dependent sorting directions of activity state space underlie recency-like phenotype. (A) Activity states of an RNN trained on 50k episodes after first three cues. Coloring by first cue (leftmost of colored PC plots), second cue (middle), and third cue (right) reveal distinct sorting directions that organize points within clusters. Sorting angles approach orthogonality in PC3 space. (B) Same as (A) for the network after 20k episodes. Activity space clusters are less distinct, and sorting angles less close to orthogonality. In particular, *θ*_12_ is more acute at 20k than 50k. (C) Independently trained networks consistently showed an increase of the angle between the first and second sorting directions, *θ*_12_, from acute toward orthogonal with increased training from 20k to 50k episodes (after 20k training, *θ*_12_ = 38.5 ± 4.9°; after 50k training, *θ*_12_ = 61.5 ± 5.9°, average over 10 independently trained RNNs; *: *p* = 0.001, one-sided paired samples *t*-test. (D) Aggregated slope vs performance curve shows recency-like phenotype at 20k training episodes. (E) Same plots as in (D) but stratified by target cue identity. E.g. cue 1 plot measures serial position curve only when cue 1 is the target cue, i.e., 1-X-X-1, X-1-X-1, X-X-1-1. Curves stratified by cue 1 and 5 show strong recency-like phenotypes, whereas those for cues 2, 3, 4 do not. **: *p* < 0.01; ***: *p* < 0.001, one-sample *t*-test. Curves for (D),(E) are averages over 10 independently trained RNNs. For (A) and (B) PCA was performed on all correct trials (97% and 83% of all trials, respectively) and top 3 PCs explain 94% and 88% of activity state variance, respectively.

We found that indeed network state was neatly organized by cue history, with each successive cue causing a directional separation in activity space. We characterized this “sorting direction” for each cue (see Methods) and found that in the space spanned by the first three PCs (accounting for >88% of variance in activity), the angles of the sorting directions approached orthogonality (for an example network: *θ*_12_ = 81° between sorting directions 1 and 2; *θ*_23_ = 62° between directions 2 and 3, and *θ*_13_ = 112° between directions 1 and 3; Fig 5A).

The same network with less experience had sorting angles less close to orthogonality (for the same network after 20k training: *θ*_12_ = 61°, *θ*_23_ = 76°, and *θ*_13_ = 130°; Fig 5B). In particular, we observed that the first sorting angle, *θ*_12_, consistently increased from an acute angle toward orthogonality during training progression from the recency-like behavior to the target-selective behavior (Fig 5C). This progression of *θ*_12_ explains the recency-like response behavior of the networks.

**Fig 6:**
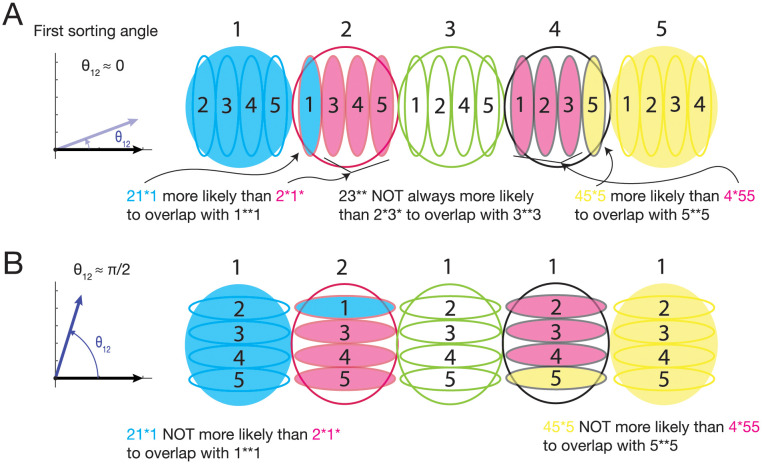
Predictions based on angle between first two cue-based sorting angles in RNN state space. *θ*_12_ is defined as the angle between the first and second sorting directions (see Methods). (A) When *θ*_12_ is small, the sorting direction of the second cue is aligned with that of the first, which manifests as cue-ordering based on second cue (smaller blobs) within clusters ordered by first cue (larger blobs) occurring in the same progression (increasing left to right). This conformation leads to particular spatial relationships, putting some clusters in closer proximity (and consequently at higher risk of overlap and erroneous readout) than others. The cluster defined by first cue 1 (big blue blob) contains the correct 1-X-X-1 match trials. Closest to this cluster is that with first cue 2 (big magenta blob). Within this cluster, the subcluster with second cue 1 is more proximal to the first cue 1 cluster than the subclusters with second cue 3, 4, or 5. This results in 2-1-X-X being more likely to overlap (and cause an erroneous match error) than 2-[3/4/5]-X-X, the former of which contains 2-1-X-1 sequences and the latter of which contains 2-X-1-1. This exactly predicts a recency-like relationship, i.e., match errors for 2-1-X-1 > 2-X-1-1. A similar pattern occurs with first cue 4 cluster’s (black large blob) subclusters’ likelihood of overlap with first cue 5 cluster (large yellow blob). In contrast, overlaps between middle first cue clusters (2 (big magenta blob), 3 (big green blob), 4(big black blob)) do not have this spatial property, and would not be predicted to exhibit a recency-like relationship. For example, 2-3-X-X is less likely to overlap with the first cue-3-cluster (large green blob) than 2-4-X-X or 2-5-X-X, but more likely than 2-1-X-X. Given the uneven match error profiles (see Fig 3B), this makes 2-3-X-3 > 2-X-3-3 match errors (i.e., the recency phenotype) very unlikely. (B) When *θ*_12_ >> 0 and closer to orthogonality, the proximity effect is removed so that match errors for X-A-X-A and X-X-A-A are equally likely across all clusters.

To illustrate this, first consider the extreme case where sorting directions 1 and 2 are the same, i.e., *θ*_12_ = 0°. In this case, as illustrated in Fig 6A, any overlap between points from the cloud defined by first cue 2 with the cloud defined by first cue 1 (large red and blue blobs in Fig 6A, respectively) would more likely be 2–1-X-X than 2-Z-X-X (where Z is any cue other than cue 1 or 2; in this case cue 3, 4, or 5). Thus, between these two points clouds, 2–1-X-1 is more likely to be mistaken for 1-X-X-1 than 2-X-1–1, which is exactly the recency-like phenotype.

This explanation makes a strong prediction based on the organization of the remaining point clouds (with first cue 3, 4, 5), namely that a similar recency-like phenotype will occur between 4–5-X-5, 4-X-5–5, and 5-X-X-5, but not so for the other cue combinations (e.g. 2–3-X-3, 2-X-3–3, and 3-X-X-3) (see Fig 6A). We found that this prediction was highly accurate. While altogether, a recency-like phenotype was apparent (Fig 5D; *p* = 8.3828e−5, one-sample Student’s *t*-test), when stratified by target cue (which measures errors made in confusion with 1-X-X-1, 2-X-X-2, 3-X-X-3, etc.) a strong recency-like phenotype was seen with target cues 1 and 5 (*p* = 0.0020 and *p* = 0.0046, respectively; Student’s *t*-test), but not with target cues 2, 3, 4 (*p* = 0.7929 and *p* = 0.2833 *p* = 0.3312, respectively; Student’s *t*-test; Fig 5E), exactly as predicted by a small *θ*_12_ ≈ 0°.

Further training led to increased cluster separation and also increased *θ*_12_ >> 0° toward orthogonality, which decreases likelihood of the recency-like phenotype (Fig 6B).

### Using recency to learn

Given our finding that RNNs exhibit the recency-like phenotype but not due to a recency-based strategy, we investigated how explicit encouragement of a recency-based strategy would alter RNN behavior, specifically if it would alter the RNNs’ solution mechanism. To do this, we altered the reward scheme of the task to explicitly promote a recency-based strategy: “match” responses to X-A-X-A and X-X-A-A trials were now rewarded, with X-A-X-A resulting in a larger reward than X-X-A-A (A-X-X-A still had the highest reward; all other rewards were unchanged; see Methods for details).

As expected, under the new reward scheme, RNNs progressed through a larger serial position curve linear fit slope deflection, reflecting an exaggerated X-A-X-A/X-X-A-A ratio (S8A Fig) indicative of recency-like behavior. Interestingly, they also progressed in performance more rapidly than with the original reward scheme.

**Fig 7:**
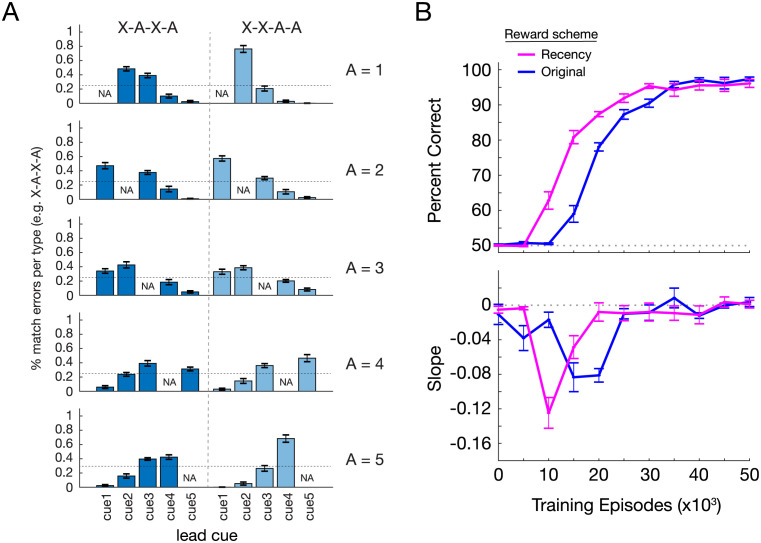
Recency-based reward scheme accelerates formation of the same solution state space organization. (A) Serial position curve error profiles reveal similar uneven and directional trends as with the original reward scheme. Error rates across different lead cues ranged from 76% to 0%, far from the even 25% predicted by a pure recency-based strategy. Shown for RNN with similar performance and behavior phenotype as that in Fig 3B (72.4% correct, 12k training trials). (B) Top, The recency-based reward scheme (magenta) leads to increases in performance earlier in training than the original reward scheme (blue). Bottom, The recency-based reward scheme causes an earlier and larger slope deflection in the expected direction during training than the original scheme. Bar and line plots represent mean of 10 independently trained RNNs. All error bars are SEM.

We checked the match error profile by lead cue, with the expectation that, unlike with the original reward scheme, RNNs with the recency-based reward scheme would have response distributions insensitive to lead cue during the period of recency-like phenotype. To our surprise we found this was not the case. Though less accentuated for the corresponding performance level in the original reward scheme (compare Figs 7A and 3 where performances are similar at 72.4% and 77.6%, respectively), error profiles were still starkly uneven (ranging from 76% to 0%), in a directionally identical manner (Fig 7A).

This suggests that since the end solution of both reward schemes is the same—reward is maximized by adopting a high target-selective strategy—recency simply speeds up the learning progression. More specifically, the recency-based reward scheme facilitates learning of the task, accelerating the solution state space transformation, albeit in the exactly the same manner (Fig 7B and S8B Fig).

### Invariance of recency-like behavior across cue encodings

One explanation for our findings thus far may be related to the method of cue input encoding. As can be observed from our prior analyses, independently trained networks exhibited a consistent state space solution after training, resulting in the easily identifiable sorting directions described above. In our simulations thus far, cues were encoded by a scalar, simulating a similarity-based coding system. For example, the five cues in the Match-First task were encoded as scalars [1–5], analogous to monkeys encoding cues in a hierarchy according to a metric of similarity perception.

**Fig 8:**
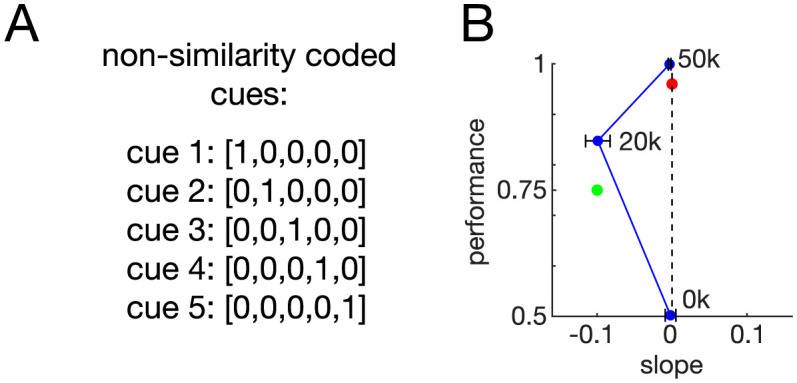
RNNs trained with non-similarity-based cue encoding also exhibit recency-like behavior. (A) An example of five cues encoded using a non-similarity based method, each equal magnitude and unrelated to the others. (B) RNNs (25 neural units, cue set size 5 cues (i.e., possible cue types), 4-step trial size) trained on Match-First with the new encoding also exhibited a recency-like behavior, seen as the negative slope of the serial position curve of intermediately trained (20k training episodes) RNNs. Further training resulted in a zero slope serial position curve (at 50k training episodes) indicative of a target-selective strategy. Error bars are SEM for 10 independently trained RNNs.

To test whether our findings were a result of the method of cue encodings, we modified the cue encodings such that they did not exhibit a similarity-based encoding, but rather were each identical in magnitude. Cues in this non-similarity-based encoding were presented as 1-hot vectors, e.g. cue 1 was represented as [1, 0, 0, 0, 0], cue 2 as [0, 1, 0, 0, 0], etc. for all five cues (Fig 8A). Interestingly, RNNs trained with this new cue encoding also exhibited the recency-like behavior, measured as a negative slope of the serial position curve (compare Figs 8B to 5D).

**Fig 9:**
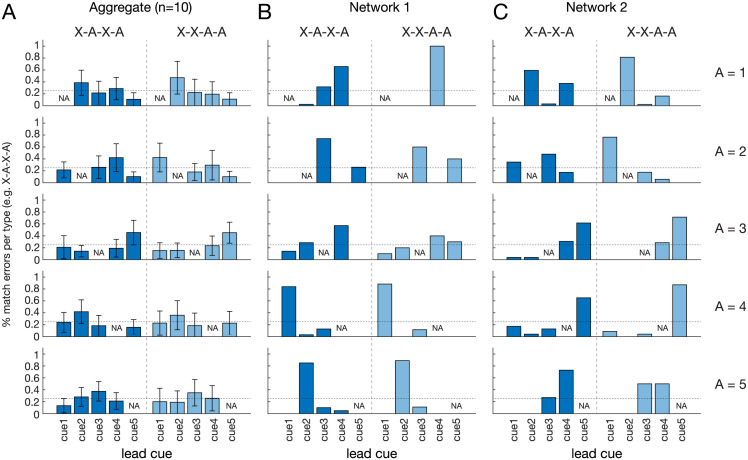
Independently trained RNNs with non-similarity-based cue encoding exhibit idiosyncratic match error profiles. (A) Match errors plot similar to Fig 3B: A in sequences shown at the top represents the cue of interest, and X represents any cue other than A, e.g. in the top row A = 1; dark blue bars indicate X-1-X-1 cue sequences and light blue bars indicate X-X-1-1 cue sequences. A identities for each row is given on the right. For RNN trained on 20k trials and exhibiting recency behavior, both dark and light bars show an even distribution of match errors across different lead cues. This parity across lead cues (all 25% of total errors (horizontal dotted line in each graph) could be suggestive of a true recency-based strategy. Error bars are 95% CI calculated from 10 independently trained RNNs. (B) and (C): Match error plots for two independently trained networks (referred to as Network 1 and Network 2) after 20k training trials. Individual RNNs have widely varying match error profiles, indicating idiosyncratic underlying network state representations. (B) Same as (A) but match errors for an individual network, Network 1. (C) Same as (B) but for Network 2.

To investigate whether this recency-like behavior was in fact recency, we again measured the error rates of mismatches separated by different lead cues as we did in Fig 3B. At first glance, the networks seemed to exhibit error profiles indicative of recency behavior, i.e., a parity of error rates (25%) indiscriminate of lead cue. Yet further investigation revealed individual networks had starkly varying error rates across lead cues (Fig 9B and 9C), similar to our previous finding which refuted a recency-based strategy. In this case, aggregate statistics of error rate profiles across independent networks simply averaged widely varying idiosyncratic behaviors such that averages appeared to show evenly distributed behaviors mirroring true recency behavior. To confirm that individual networks were exhibiting strongly variant mismatch error profiles, akin to that seen in the previous analysis with the similarity-based cue encoding, we investigated the error profiles of two example networks (called Network 1 and Network 2 for these analyses).

If the variation in the mismatch error profile were a product of state space clustering geometry as was the case for the similarity-based cue encoding networks, higher error rates should indicate overlapping clusters of state space representations of particular cue sequences which can be readily predicted. For Network 1, the error rate profile shown in Fig 9B suggests the network state space representations for sequences beginning with cue 1 are closest to the state space representations of sequences beginning with cue 4 (indicated by the large % match error per type when cue 1 is the target cue (top row) and cue 4 is the lead cue), i.e., 4-1-X-1 is more likely to cause an error (mistaken for 1-X-X-1 which would be correct) than 3-1-X-1, 2-1-X-1, or 5-1-X-1. Similarly we predicted that the state representations of sequences beginning with cue 2 would be closest to those of cue 5 (see bottom row of Fig 9B where target cue is cue 5 and largest error rate occurs when cue 2 is the lead cue).

**Fig 10:**
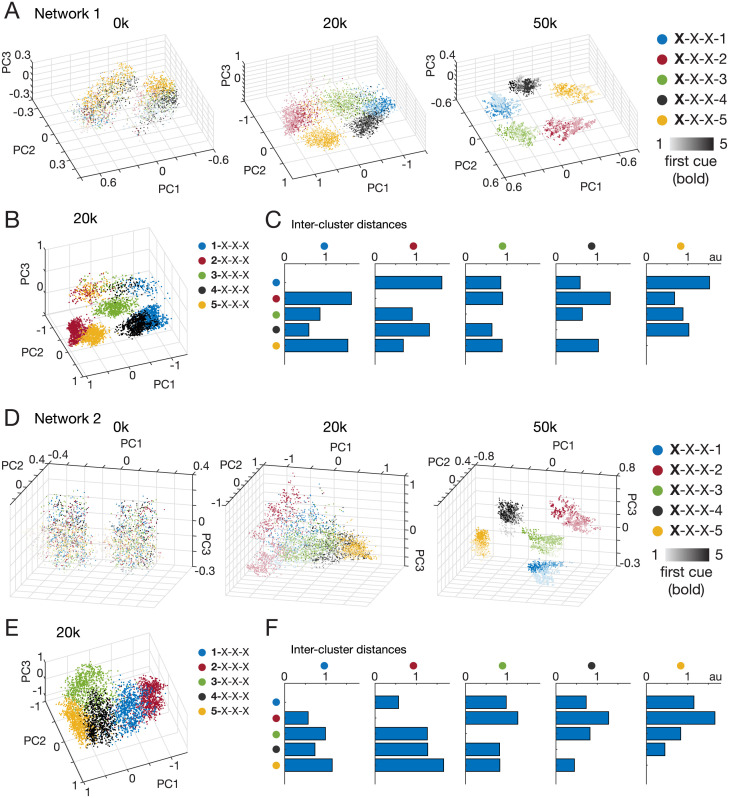
Independently trained RNNs with non-similarity-based cue encodings have unique state representation geometries with intercluster distances predicted by mismatch error rates. (A) PCA of network activity after presentation of all cues in a trial for trials answered correctly, colored by last cue (target cue) and shaded by first cue in sequence. The untrained RNN (0k) outputs random behavior, at 20k training episodes, distinct match and no match clusters begin to form with overlapping borders, and at 50k, clearly demarcated clusters in activity space are apparent. For Network 1, the clusters (by target cue) formed a circular geometry. (B) PCA of activity states of an RNN trained on 20k episodes after first three cues, colored by first cue. Centroids of each cluster were calculated. (C) Intercluster distances measured from centroids of each cluster. Colored dots on axes represents cluster identity from legend shown in (B), with distances measured in arbitrary units (au). Intercluster distances were predicted by mismatch error rates for Network 1 from Fig 9B. (D) Same as (A) but for a different network, Network 2. The clusters (by target cue) formed a non-circular geometry, different from Network 1’s geometry. (E) Same as (B) but for Network 2. Orientation of clusters is different from Network 1. (F) Same as (C) but for Network 2 with cluster identity key shown in (E). Intercluster distances are different than for Network 1 and were predicted by mismatch error rates for Network 2 from Fig 9C. Top 3 PCs explained >72% of activity variance for all analyses.

Due to the non-similarity based encoding of cues, independently trained networks could result in completely different network solution state space geometries, as seen in comparing the solution state spaces of Network 2 to Network 1 (Fig 10A and 10D). For Network 2, using the same logic as described above for Network 1, we predicted the network state representations for sequences beginning with cue 1 would be closest to those of sequences beginning with cue 2, and the state representations of sequences beginning with cue 4 would be closest to those of cue 5.

Next, we checked whether the state representations of the individual networks matched our predictions of state space representation geometry. As can be seen in Fig 10A and 10D, independently trained networks formed idiosyncratic state representations. During training, clusters of state representations separable by target cue identity began to form and then became well-separated (Fig 10A and 10D), much as previously seen for the similarity-based cue encoding trained networks. To test our predictions of state space representation geometry, we calculated the centroid of each state representation cluster identified by the lead cue (exactly the same as done in Fig 5A and 5B). We then measured the inter-cluster distances by calculating the distance between cluster centroids. We found that our predictions were correct: for Network 1, the cluster of state space representation for sequences with lead cue 1 was closest to that of lead cue 4 (see leftmost barplot of Fig 10C), and the cluster for lead cue 2 was closest to that of lead cue 5 (see rightmost barplot of Fig 10C). Our predictions for Network 2 were also correct: the cluster for lead cue 1 was closest to that of lead cue 2 (see leftmost barplot of Fig 10F), and the cluster for lead cue 4 was closest to that of lead cue 5 (see rightmost barplot of Fig 10F). To verify this relationship held true consistently, we calculated the correlation between pairwise intercluster distances as described above (e.g. between the cluster for 1-X-X-X and the cluster for 2-X-X-X), and corresponding pairwise match error rates (e.g. error yes response rate of 2-1-X-1 and 1-2-X-2 type trials). We did this for each independently trained RNN (n=10). We found that all RNNs exhibited a moderate to strong inverse correlation, with Pearson correlation coefficients ranging from *r* = -0.54 to *r* = -0.80, and all correlations were statistically significant at an *α* = 0.05 ranging from *p* <1e-4 to *p* = 0.0137. In aggregate over all RNNs, the mean correlation coefficient was *r* = -0.68 and mean significance was *p* = 0.003, showing that smaller intercluster distances consistently predict higher match error rates.

These results show that the recency-like behavior observed of RNNs trained on the Match-First task was invariant across different methods of cue-encoding. Furthermore regardless of cue encoding method, the recency-like behavior was a result of overlapping state space representations resulting in very specific types of mismatch errors rather than a simple recency mismatch behavior. Thus, while it is unknown how monkeys encode the cues of the task,—whether using a similarity-based encoding or a non-similarity-based encoding—in either case the recency-like phenotype may be due to overlapping neural network state space representations of sequences as is the case for our RNN models, rather than a true recency-based strategy.

## Discussion

In following up Wittig et al.’s study [8], we uncovered two major unexpected findings: 1) what appears to be recency behavior may not in fact be true recency; and 2) in spite of this, a recency-based strategy may facilitate learning of the non-recency strategy progression. Focusing on state space solutions elucidated how these surprising results fit together in a coherent model of learning; one that may explain what appears to be different strategies used by monkeys and humans.

Our results support the hypothesis put forward by Wittig et al. [8] that a major component driving the differences observed of humans and monkeys on the working memory tasks may be task understanding (accrued from number of experiences of the task), which we show can further be influenced by the capacity of the network dedicated to learning the task. Our finding suggests humans and monkeys may lie on two different points of the same learning progression. Thus, the apparent differences in strategies can be considered a consequence of development. The hypothesis of “differential use of language” mentioned by Wittig et al. [8] was not tested in our work. Our network models show that even without the contribution of language, the behaviors of monkeys and humans can be reproduced. Language likely acts to rapidly accelerate task understanding, expediting acquisition of strategies observed of highly experienced networks.

RNNs trained to high task proficiency had activity trajectories suggestive of learned ignoring of distractor cues, yet deeper analysis revealed high-fidelity encoding of distractor cues as well as relevant cues. This finding follows the observations of Marshall et al. and others that task irrelevant features are often encoded even when subjects explicitly understand their irrelevance [29]. In our analysis, activity space organization is shaped by task irrelevant cues (distractor cues) (our observations of sorting directions and intercluster distances) which causes the observed recency-like behavior. Our results suggest that task irrelevant feature encoding rather than simple repetition may drive this observed behavior.

The finding that a recency-reward scheme facilitated attainment of the optimal target-selective strategy, raises an interesting possibility that some strategies might be useful simply to accelerate learning. Such strategies may themselves not be optimal, but act as a “catalytic strategy,” helping shape the structure of the state space in a way that expedites achievement of the optimal solution. This concept is similar to “shaping,” in which intermediate training goals are used to piece together a larger, often multi-step behavior. Catalytic strategies could then function as a type of internal shaping; for example, a monkey that derives some positive feeling from incorrect recency trials (perhaps the familiarity of seeing a number repeat) would get to optimal performance faster than a monkey zealously following the external reward program. Identifying catalytic strategies—perhaps within the vast repertoire of heuristics humans use—promises to uncover new insights into the mechanisms underlying cognitive processing.

Our modeling work makes a number of predictions that can be tested:

1) In monkeys, is the match error profile across specific lead/distractor cues uniform as predicted by a pure recency-based strategy, or is it sharply varying as predicted by our RNN model? If the distribution is uniform, this suggests a true recency-based strategy, perhaps relating to prior experience or shaping behaviors not captured in our models, which lack the rich experiential history of organisms like humans and monkeys. If the distribution is sharply varying, this might indicate that monkeys follow the developmental trajectory taken by our network model, showing a recency-like phenotype that in fact is not pure recency-based.
2) Our model predicts that using a recency-based reward scheme will speed up learning of the task. Adding this explicit recency-rewarding protocol during training of monkeys and comparing them to monkeys trained with the normal reward scheme can test this prediction.
3) Our RNN models predict a particular solution state space organization for the working memory tasks. This solution state space result was robust, occurring repeatedly for independently trained networks, suggesting a solution for the task that is potentially optimal for systems based on networks. Collection of large-scale neural recordings from monkeys (e.g. regions of prefrontal cortex thought to be critical to solving these working memory tasks) during and after training on the tasks (e.g. Match-First, set size 5) will allow assessment of how well the RNN model’s solution state space matches that of monkeys neural circuits. Furthermore, collection of such data would allow differentiation between similarity-based and non-similarity-based cue encoding by showing either consistency of state space representation geometry across different animals (as in similarity-based encoding) or idiosyncratic activity state clustering (as in non-similarity based encoding).

Our findings confirm the central message of Wittig et al. 2016: that extrapolation from one model system to another should be done with caution rather that assuming similarity based on coarse-grain measures. Wittig et al. showed monkey and human behaviors have different characteristic features even though performance may be comparable; here we show that the explanation for that difference may not be the most obvious, and that although they are different, monkeys and humans may lie on different points of the same learning continuum.

Finally, we note the inherent and unavoidable challenges of comparing model systems—humans with monkeys, and RNNs with both—and emphasize the importance of validation studies to assess findings and predictions from one model system to another. Just as animal models are useful to study how humans brains operate, RNNs are a powerful model system for studying neural computations of biological neural networks most notably in enabling full access for analysis of all individual and populations of neural units that make up the network as well as the ability to readily and at scale vary key network parameters and study the effects of such variation [13–22]. Yet monkeys have very different life histories than the humans they are compared to, and likewise, RNNs have, even more starkly, no life histories prior to task introduction. Each system has architectural differences, and our RNN models adopt a common basic model to gain insight into both monkey and humans, ignoring these architectural differences aside from variation in number of neural units. In the lab, monkeys must go through rigorous shaping protocols over the course of several months to successfully train them on experimental tasks. These include components as simple as sitting still or fixating at a point on a screen consistently. Humans in contrast can be told the task and tested within a single day of training, inferring many of the implicit protocols of the experimental assessment (e.g. chair in the room likely means sit; screen likely is the place to look; language guidance explains the task). RNNs have an inductive bias regarding the task even before training begins, as do humans and monkeys. The extent to which the inductive bias of RNNs is similar to those of brains makes them useful in understanding the others, but their fundamental differences must be acknowledged in the analyses that extrapolate from one to the others.

## Methods

### Working memory tasks from Wittig et al. 2016 [8]

Our analyses were done with the working memory tasks used by Wittig et al. (described in the first Results section; see [8, 12] for the all details). In brief, there were three tasks: Same-Different (SD), in which a response indicates if the second cue matches the first cue; Match-First (MF), in which a response indicates if the last cue matches the first cue in a cue sequence; and Match-Any (MA), in which a response indicates if the last cue matches any of the previous cues in the current trial. Each task had variations depending on the set size (number of different cues that could be selected: 2 or 100 for SD, 5 or 100 for MF and MA) and trial size (number of steps in a single trial: always 2 for SD; 4 or 8 for MF and MA), leading to a total of 8 task variations tested (SD 2 or 100 cue; MF set size 5, trial size 4; MF set size 100, trial size 4; MF set size 100, trial size 8; MA set size 5, trial size 4; MA set size 100, trial size 4; MA set size 100, trial size 8). We computationally implemented each of these task variations and trained and tested all network configurations on each task.

Cues were encoded as a single value ranging from [1,maximum cue] (e.g. for Match-First, set size 5, this was [1..5]; in the last section of Results this is referred to as the similarity-based coding system). For timesteps during which no cue was presented, [0] was presented in place of any cue. Trials were separated by a timestep with no cue presented. For example, two trials of Match-First, with trial size 4, and set size 5, would be presented as:

$$\begin{array}{c} 0-3-1-5-2-0-2-4-3-2-0 \end{array}$$

Rewards were given after the RNN’s decision of “match” or “no match” following presentation of the final cue in a trial (e.g. after the first 2 and after the last 2 in the sequence shown above—correct answers would be “no match” and “match,” respectively). Correct trials were rewarded with +5 and incorrect trials with -5. At all other timepoints reward feedback was 0.

In the recency-based reward scheme for Match-First, two modifications were made to encourage recency-based behavior. X-A-X-A trials in which the RNN responded “match” were rewarded +3 and X-X-A-A trials in which the RNN responded “match” were rewarded +1 (in original reward scheme reward for both these would be -5). All other rewards remained unchanged.

In the last section of the Results we also trained networks using a non-similarity-based cue encoding. Cues were coded as 1-hot vectors (e.g. for Match-First, set size 5, cue 1 was presented as [1, 0, 0, 0, 0], cue 2 as [0, 1, 0, 0, 0], cue 3 as [0, 0, 1, 0, 0], cue 4 as [0, 0, 0, 1, 0], and cue 5 as [0, 0, 0, 0, 1]. For timesteps without a cue (i.e., between trials), a zero vector was presented ([0, 0, 0, 0, 0]). The RNNs were adjusted to accommodate cue input of this form. Otherwise all simulations were run in an identical manner to those described previously. Below is an example of how two trials of Match-First with trial size 4 and set size 5 would be presented:

[0, 0, 0, 0, 0]—blank screen
[0, 0, 1, 0, 0]—cue 3
[1, 0, 0, 0, 0]—cue 1
[0, 0, 0, 0, 1]—cue 5
[0, 1, 0, 0, 0]—cue 2
[0, 0, 0, 0, 0]—blank screen
[0, 1, 0, 0, 0]—cue 2
[0, 0, 0, 1, 0]—cue 4
[0, 0, 1, 0, 0]—cue 3
[0, 1, 0, 0, 0]—cue 2
[0, 0, 0, 0, 0]—blank screen

### Recurrent neural networks models

Our RNN models consisted of LSTMs of different sizes (*n* ∈ {5, 10, 25, 50, 100, 200, 500} neural units). Unlike “vanilla” RNNs, LSTMs incorporate multiple intrinsic gates that influence the RNN’s state dynamics with respect to retaining information, incorporating new information, and outputting information [30]. This gating structure enables robust learning and behaviors mimicking animal and human models on multiple tasks [16, 18]. The LSTM states and gates are described by the following equations:

$$\begin{array}{cc} f_{t} & =\sigma (W_{xf}x_{t}+W_{hf}h_{t-1}+b_{f})\\ i_{t} & =\sigma (W_{xi}x_{t}+W_{hi}h_{t-1}+b_{i})\\ o_{t} & =\sigma (W_{xo}x_{t}+W_{ho}h_{t-1}+b_{o})\\ c_{t} & =f_{t}\circ c_{t-1}+i_{t}\circ tanh\left(W_{xc}x_{t}+W_{hc}h_{t-1}+b_{c}\right)\\ h_{t} & =o_{t}\circ tanh\left(c_{t}\right) \end{array}$$

where *f*_*t*_, *i*_*t*_, and *o*_*t*_ are the forget, input, and output gates at time *t* respectively, *σ* is the sigmoid activation function, *W*_*ij*_ denotes the weights from component *i* to component *j*, *x*_*t*_ is the external input at time *t*, *h*_*t*_ is the ouput of the LSTM at time *t*, *c*_*t*_ is the state of the LSTM cell at time *t*, *b*_*f*_, *b*_*i*_, and *b*_*o*_ are the biases of the forget, input and output gates respectively, *b*_*c*_ is the bias of the cell states, and ∘ denotes the Hadamard product.

**Fig 11:**
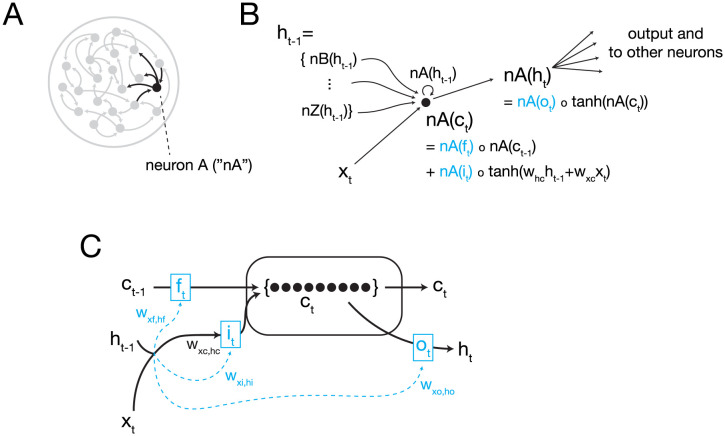
Schematic of LSTM. (A) An LSTM is a type of RNN (greyed neural network; dots represent neurons, arrows represent connections) with intrinsic gating to support robust memory [30]. To illustrate how the LSTM operates, a single example neuron, “neuron A,” is highlighted and its governing equations are shown in (B) (black neuron and input and output connections). (B) Neuron A, abbreviated nA here, takes input from the prior output of the network (*h*_*t*−1_) (hence the ANN’s recurrent nature) and external input (*x*_*t*_, e.g. a visual cue). *h*_*t*−1_ is a vector made of up input for each individual neuron in the network into neuron A, including itself (*nA*(*h*_*t*−1_)), input from neuron B (*nB*(*h*_*t*−1_)), input from neuron Z (*nZ*(*h*_*t*−1_), etc. The current state of neuron A (*nA*(*c*_*t*_)) is calculated by the sum of the product of the forget gate (*nA*(*f*_*t*_)) and the previous state (*nA*(*c*_*t*−1_)) and product of the input gate (*nA*(*i*_*t*_)) and the weighted inputs to the network (see equations, where light blue indicates a gate function, e.g. *nA*(*f*_*t*_)) and ∘ denotes the Hadamard product. Output *nA*(*h*_*t*_) is calculated from the output gate *nA*(*o*_*t*_) applied to the current state, which is then input at the next time step for downstream neurons. Bias terms are omitted in the schematic. (C) A schematic of a whole LSTM network, with dots representing individual neurons’ activity states and light blue boxes representing intrinsic gates. Inputs to the network (*h*_*t*−1_ and *x*_*t*_) modulate the forget, input, and output gates (*f*_*t*_, *i*_*t*_, *o*_*t*_, respectively) through weights (forget gate weights: *w*_*xf*, *hf*_; input gate weights: *w*_*xi*, *hi*_, output gate weights: *w*_*xo*, *ho*_. Weighted sums of inputs (input weights: *w*_*xc*, *hc*_) are input gated and added to the forget gated state of the network from the prior timestep (*c*_*t*−1_) to determine the new state of the network (*c*_*t*_). Output gating (*o*_*t*_) of the current network state generates network output (*h*_*t*_). Sigmoid and hyperbolic tangent operations are ommited from schematic.

Intuitively, LSTMs are RNNs with weights that modulate how much of prior network activity is persevered, how much new input activity is incorporated, and how much network activity is output. The RNN has specific weights that modulate input, including both new external stimuli (e.g. a new cue presentation) and input of the network’s prior state (i.e., a neuron’s recurrent input into another neuron in the RNN) (input gate); weights that modulate forgetting of previous neuron activity (which can be thought of as degradation or preservation of a neuron’s activity over time); and weights that modulate outputs (output gate) (Fig 11). This form of gated RNN permits flexible learning over relatively long time-scales more robustly than an ungated “vanilla” RNN [30].

### Reinforcement learning

We trained our RNNs by reinforcement learning [31], similar to how humans and animals learn many tasks such as the working memory tasks from Wittig et al. 2016. Similar to [18], we used an Advantage Actor-Critic algorithm (see Mnih et al. [32] for a full description of the algorithm). In brief, the objective function consists of the gradient of a policy term, an advantage value term, and an entropy regularization term:

$$\begin{array}{cc} \nabla L & =\nabla L_{\pi }+\nabla L_{v}+\nabla L_{H}\\ & =\frac{\partial log\pi (a_{t}|s_{t};\theta )}{\partial \theta }\delta_{t}\left(s_{t};\theta \right)+\beta_{v}\delta_{t}\left(s_{t};\theta \right)\frac{\partial V}{\partial \theta }+\beta_{H}\frac{\partial H(\pi \left(a_{t}|s_{t};\theta \right))}{\partial \theta } \end{array}$$

where *π* is the policy output by the RNN (passed through a softmax function), *a*_*t*_ is the action taken at time *t*, *s*_*t*_ is the state at time *t*, *θ* is the network parameters, *β*_*v*_, *β*_*H*_ are hyperparameters for scaling the contribution of the value and entropy terms respectively (we set *β*_*v*_ = *β*_*H*_ = 0.05 for all our simulations), *V* is the value output of the network, and *H* is the entropy regularization term of the policy. The softmax function for the policy term is given by:

$$\begin{array}{c} \pi \left(a_{t}|s_{t};\theta \right)=\frac{e^{h(s_{t},a_{t},\theta })}{\sum_{b}e^{h(s_{t},b_{t},\theta })} \end{array}$$

*δ*_*t*_ is the advantage estimate, which represents the temporal difference error:

$$\begin{array}{c} \delta_{t}\left(s_{t};\theta \right)=R_{t}-V\left(s_{t};\theta \right) \end{array}$$

where *R*_*t*_ is the discounted reward:

$$\begin{array}{c} R_{t}=\sum_{i=0}^{k-1}\gamma^{i}r_{t+i}+\gamma^{k}V\left(s_{t+k};\theta \right) \end{array}$$

where *k* is the number of steps until the next end state. When *γ* = 0, *R*_*t*_ = *r*_*t*_.

The advantage equation in this case is equivalent to a temporal-difference error signal enabling temporal difference reinforcement learning.

The parameters of the model were updated during training by gradient descent and back propagation through time using AdamOptimizer with learning rate 1*e* − 03. Training occurred after every 3 trials. For all tasks, we set *γ* = 0 since actions at time points prior to the decision timepoint had no bearing on outcome.

Similar to the implementation of others [16, 18], input to networks at each time point was given as vector with the current cue, the action taken at the previous time point, *a*_*t*−1_, and the reward received at the previous timepoint, *r*_*t*−1_.

RNNs were trained for 50,000 trials (*n*_*ep*_) and tested throughout after *n*_*ep*_ ∈ {0, 5, 10, 20, 30, 40, 50} x 10^3^ trials. To compare 25 unit RNNs on Match-First, set size 5 with the original and recency-based reward schemes, we trained RNNs with either reward scheme and tested every 5 x 10^3^ trials. To compare RNN activity state spaces across reward schemes we selected training points with similar performance (and close to that of monkeys on the task) which was 20k for the original (77.6% correct) and 12k for the recency-based reward scheme (72.4% correct).

### Serial position curves

Following Wittig et al. 2016 we measured serial position curves for RNNs. Serial position curves give the fraction of yes (i.e., “match”) responses when a cue at a given position matches the target cue (last cue in a trial). For example, for Match-First, trial size 4, if the target cue was cue 1, the serial position curve would give the fraction of yes/no responses when cue 1 was in the first position (-3 test recency position in Fig 3 of Wittig et al. 2016; yes response in this case was a correct match), fraction of yes/no responses when cue 1 was in the second position (-2 test recency position), and same for third position (-1 test recency position). These fractions (e.g. -2 and -3 test recency positions for Match-First, trial size 4) then can be fit with a least-squares linear fit as was done in Fig 3 of Wittig et al. 2016 [8]. The linear fit can be used to calculate a residual that describes the deviation of the observed “hit rate” (yes/no fraction at -3 test rececny position) from the linear fit; this is what we refer to as the linear fit residual. The slope of the linear fit indicates the presence or absence of a recency-like behavior; this is what we refer to as the linear fit slope.

It should be noted that the least-squares linear fit of the serial position curves is an approximation of the trend in response frequency across positions. Several variations of this approximation can be used, for example including more or fewer positions for the linear fit (in our analyses we used the two adjacent positions as was done in Wittig et al. 2016 [8] unless otherwise noted), or using different fit functions like a sigmoid. Whichever method of approximation is used, some edge cases result in less useful metrics. Two examples of this include if the positions used for the fit result in an incredibly positive or negative slope, in which case the linear-fit residual may be nonsensically large compared to measurements in other situations; another example is if there is no clear trend across positions, but making a linear fit across some subset of positions gives a random trend. These misleading cases due to intrinsic noise in the data is are controlled through high numbers of replicates of independently trained networks (equivalent to multiple subjects in experiments) and through high numbers of sample replicates from any given network/subject, both as indicated in the text and Methods above.

### Sorting directions

Sorting directions in Fig 5 were computed by calculating the centroid of each point cloud in the space of the first three principal components (which accounted for 94% and 88% variance explained for RNNs trained for 50k and 20k trials for similarity-based cue encoding simulations, respectively) identified by the cue presented at a given timestep in the trial. A least-squares error line was fit to these centroids to give the sorting direction. This was calculated for network states after being presented with the first 3 cues in the sequence for Match-First on all correct trials from 5000 test trials at each level of training (50k training episodes for Fig 5A, and 20k for Fig 5B). The first sorting direction was calculated with respect to the first cue presented (different colors in Fig 5A and 5B leftmost colored PC plots). The second and third sorting directions were calculated using the point cloud in which the first cue was cue 1 (i.e., within the blue point cloud in the leftmost colored PC plots in Fig 5).

### Intercluster distances

Intercluster distances in Fig 10 were computed by calculating the centroid of each point cloud in the space of the first three principal components (which accounted for >72% of variance explained across all analyses shown with non-similarity-based cue encoding simulations) identified by the lead cue presented (Fig 10B and 10E). Pairwise distances between centroids was calculated. This was calculated for network states after being presented with the first 3 cues in the sequence for Match-First on all correct trials from 5000 test trials after 20k training episodes.

## Supporting information

S1 Fig Performance over training episodes for RNNs across a size spectrum. Performance over eight variations of three working memory tasks from Wittig et al. 2016. Across all tasks, smaller networks (lighter blue colors) required more training episodes to achieve similar performance to larger networks (darker blue colors). For some tasks, networks across the whole size spectrum could gain proficiency (Same-Different, set size 2), for others only some networks reached maximum proficiency by the maximum training duration (e.g. Match-First, set size 5), and for others no network reached near perfect performance (e.g. Match-Any, set size 5). Similarly to humans and monkeys, RNNs performed the worst on Match-Any. All performance curves are the average performance of 10 independently trained networks of the given size, tested with 5000 test episodes at each training point. Error bars represent standard error over 10 independent networks tested.
(EPS)

S2 Fig Serial position curve linear fit residuals on all task variations from Wittig et al. 2016. Residuals of serial position curve linear fits (linear fit residual) as described in Wittig et al. 2016 closely mimic performance of RNNs on each task. A linear fit residual of 1 represents a pure target-selective behavior whereas a value of zero represents a pure non-selective strategy. For each task variation, the correlation coefficient between linear fit residual and performance at each network configuration (size x training) is given; all are upwards of 0.9 except Match-Any, set size 100 (R = 0.4) which the RNNs had trouble learning in the training duration. All linear fit residual curves are the average of 10 independently trained networks of the given size, tested with 5000 test episodes at each training point. Error bars represent standard error over 10 independent networks tested.
(EPS)

S3 Fig Two possible modes of strategy evolution. Schematics of two hypothetical strategy evolution scenarios shown for the Match-First task. For each part, left shows the progression of the serial position curve, right top shows the performance vs linear fit residual, and bottom right shows performance vs linear fit slope. (A) Strategy progression from random to increasingly target selective. Behavior begins as random with uniform yes rate across serial positions, then yes rate progressively increases at target location (“hit rate”) and yes rate at all other positions (“false alarm rate”) decreases uniformly. Residual of linear fit will increase with increasing performance and slope will trace random walk around 0. (B) Strategy progression from random to recency-like to target-selective. From initial random behavior, yes rate at positions close to target location increase (or decrease more slowly) relative to yes rate at further positions, exhibiting recency-like behavior. Then hit rate continues to increase, while false alarm rate becomes uniform and low in a target-selective strategy. Linear fit residual initially remains near 0 with initial increase in performance during recency-like behavior, then increases with further performance improvement. Slope beings at 0, deflects in an expected direction (negative for Match-First) then returns to zero when target-selective strategy is achieved.
(EPS)

S4 Fig Serial position curve linear fit slope and residual for all task variations from Wittig et al. 2016. Slope and residual plots reveal clear instances where RNNs progress from random (low performance, small slope, small residual) to recency-like (higher performance, slope deflected in predicted (green) direction, low residual) to target-selective behavior (higher performance, small slope, high residual), for example Match-First, set size 5. Other RNNs did not have apparent recency-like behavior, though caveats on sampling depth and frequency require further investigation (e.g. Match-First, set size 100, distractors 2; Same-Different, set size 2; S5 Fig). In each slope plot, shaded green region indicates prediction prior on direction of slope deflection if recency-like behavior occurs. Dotted line in slope plots indicates 0 slope. Dotted line in residual plots connects from random (0 performance, 0 slope) to point achieved by human subjects in Wittig et al. 2016 (red dot). Human and monkey behavior is shown in all plots as red and green dots, respectively.
(EPS)

S5 Fig Undersampling depth and frequency masks occurrences of recency-like behavior by RNNs. (A) Example of undersampling depth. Serial position curve is constructed based on occurrence of error trials with particular cue arrangements. Task variations with large cue set size (100 vs 5 cues; 20x difference) require more sampling. Increased test sampling depth (5000 → 30000 → 100000 test samples) of same network reveals recency-like behavior when none was apparent at lower test sampling depth (5000 test samples). (B) Recency-like behavior could also be masked by sampling frequency for networks/tasks that rapidly progressed from random to target-selective strategy. Increased sampling frequency (training points ∈{0, 11, 20, 40, 50} → {0, 5, 11, 15, 20, 30, 40, 50} → {0, 5, 7, 9, 11, 13, 15, 20, 30, 40, 50}x10^3^) reveals recency-like behavior when none was apparent at lower frequency sampling.
(EPS)

S6 Fig RNN population activity trajectories on Match-First trials. Network activity trajectories on test trials for three levels of training (trained on 0k, 20k, 50k trials). Colored points indicate network state after presentation of indicated cue in the trial sequence. Untrained networks (0k) do not exhibit any apparent ordering, with network states at different timepoints in different trials mixed up. Highly trained networks (50k) show precise trajectory shape and network state organization, with relevant cues (1st and 4th) moving network state in a direction apparently different than distractor cues (2nd and 3rd). Intermediately trained networks (20k) exhibit sequential ordering of network states without showing clear directional difference by specific cue position. For visualization and comparison purposes, all plots show random 100 trajectories aligned at the trial start for correct trials that occurred following a correct trial (“correct-after-correct”); previous trial outcome had a large binary influence on activity variability. For 50k trained RNNs, these made up 97% of correct trials, which in turn made up 97% of all trials. For 20k, this was 83% and 83%; and for 0k this was 51% and 51%. Top 3 PCs on all “correct-after-correct” trials explained >85% of variance in activity at each of the three training points assessed.
(EPS)

S7 Fig Consistent topology of network solution state space across indpendently trained RNNs. (A) and (B): PCA of network activity after presentation of all cues in a trial for RNNs trained on 50k training episodes on the Match-First task. Each row represents a different, independently trained RNN. Examples of 5 independently trained RNNs are shown here (5 rows). (A) PCA of network activity after presentation of all cues in a trial for correct trials, colored by match (blue) or no match (red) identity. Clearly demarcated clusters in activity space are apparent, defining a “match” band (blue region) within a surrounding “no match” zone (red region) in very similar fashion across networks (rows). (B) Same plots as (A) but colored by last cue (target cue) and shaded by first cue in sequence. Topological arrangement is preserved across networks, specifically the proximity of particular activity clusters to others. For example, for each RNN, the dark yellow cluster (5-X-X-5) is always adjacent to the second darkest black cluster (4-X-X-4) and far from the lightest blue cluster (1-X-X-1). Each cluster (identified by color and shade) corresponds to a unique lead and last cue combination. Top 3 PCs explained >92% of activity variance across all plots shown.
(EPS)

S8 Fig Recency reward scheme trained RNNs progress from recency to target-selective behavior and learn faster. (A) Slope vs performance plot for RNNs trained with a reward scheme that explicitly rewards recency behavior compared to original reward scheme. RNNs trained with recency scheme show the same slope vs performance progression, but appear to have an accentuated recency behavior at intermediate training (more negative slope) and move through the learning progression faster (compare recency reward scheme trained RNNs slope at 10k training trials to original at 15k). Labels in graph indicate trials trained on (e.g. 15k indicates RNN has been trained on 15e3 trials). Data shown is for 10 independently trained networks in each reward scheme. Error bars are SEM. (B). Example RNN’s solution space during learning under recency reward scheme. The solution space of RNNs trained with the recency reward scheme follows a similar progression as those trained with the original scheme (Fig 4A and 4B), with a very similarly arranged solution space map after extensive training and attainment of a target-selective strategy (50k training trials). The progression occurs faster with recency reward scheme training, with performance and solution space after 12k training trials very similar to those of RNNs under the original scheme at 20k training trials. Top 3 PCs explained >88% of activity variance for all conditions.
(EPS)

## References

[1] Ericsson AC, Crim MJ, Franklin CL. A Brief History of Animal Modeling. Missouri Medicine. 2013;110:201–205. 23829102 PMC3979591

[2] Feng G, Jensen FE, Greely HT, Okano H, Treue S, Roberts AC, et al. Opportunities and limitations of genetically modified nonhuman primate models for neuroscience research. Proceedings of the National Academy of Sciences. 2020;117(39):24022–24031. doi: 10.1073/pnas.2006515117 32817435 PMC7533691

[3] Bernardi S, Salzman CD. The contribution of nonhuman primate research to the understanding of emotion and cognition and its clinical relevance. Proceedings of the National Academy of Sciences. 2019;116:26305–26312. doi: 10.1073/pnas.1902293116 PMC6936419 31871162

[4] Roberts AC, Clarke HF. Why we need nonhuman primates to study the role of ventromedial prefrontal cortex in the regulation of threat- and reward-elicited responses. Proceedings of the National Academy of Sciences. 2019;116:26297–26304. doi: 10.1073/pnas.1902288116 PMC6936360 31871181

[5] Verdier JM, Acquatella I, Lautier C, Trouche GDS, Lasbleiz C, Mestre-Frances N. Lessons from the analysis of nonhuman primates for understanding human aging and neurodegenerative diseases. Frontiers in Neuroscience. 2015;9:64. doi: 10.3389/fnins.2015.00064 25788873 PMC4349082

[6] Lutz CK. Stereotypic Behavior in Nonhuman Primates as a Model for the Human Condition. Institute for Laboratory Animal Research Journal. 2014;55:284–296. 10.1093/ilar/ilu016 PMC4240438 25225307

[7] Leenaars CHC, Kouwenaar C, Stafleu FR, Bleich A, Ritskes‑Hoitinga M, Vries RBMD, et al. Animal to human translation: a systematic scoping review of reported concordance rates. Journal of Translational Medicine. 2019;17:223. doi: 10.1186/s12967-019-1976-2 31307492 PMC6631915

[8] Wittig JHJ, Morgan B, Masseau E, Richmond BJ. Humans and monkeys use different strategies to solve the same short-term memory tasks. Learning and Memory. 2016;23:644–647. doi: 10.1101/lm.041764.116 27918285 PMC5066608

[9] Elmore LC, Ma WJ, Magnotti JF, Leising KJ, Passaro AD, Katz JS, et al. Visual Short-Term Memory Compared in Rhesus Monkeys and Humans. Current Biology. 2011;21:975–979. doi: 10.1016/j.cub.2011.04.031 21596568 PMC4634532

[10] Penn DC, Povinelli DJ. On the lack of evidence that non-human animals possess anything remotely resembling a ‘theory of mind’. Philosophical Transactions of the Royal Society B. 2007;362:731–744. doi: 10.1098/rstb.2006.2023 17264056 PMC2346530

[11] Cáceres M, Lachuer J, Zapala MA, Redmond JC, Kudo L, Geschwind DH, et al. Elevated gene expression levels distinguish human from non-human primate brains. Proceedings of the National Academy of Sciences. 2003;100(22):13030–13035. doi: 10.1073/pnas.2135499100 14557539 PMC240739

[12] Wittig JHJ, Richmond BJ. Monkeys rely on recency of stimulus repetition when solving short-term memory tasks. Learning and Memory. 2014;21:325–333. doi: 10.1101/lm.034181.113 25171424 PMC4024622

[13] Russo AA, Bittner SR, Perkins SM, Seely JS, London BM, Lara AH, et al. Motor Cortex Embeds Muscle-like Commands in an Untangled Population Response. Neuron. 2018;97:953–966. doi: 10.1016/j.neuron.2018.01.004 29398358 PMC5823788

[14] Roach JP, Churchland AK, Engel TA. Choice selective inhibition drives stability and competition in decision circuits. Nature Communications. 2023;14(147). doi: 10.1038/s41467-023-35822-8 36627310 PMC9832138

[15] Mante V, Sussillo D, Shenoy KV, Newsome WT. Context-dependent computation by recurrent dynamics in prefrontal cortex. Nature. 2013;503:78–84. doi: 10.1038/nature12742 24201281 PMC4121670

[16] Wang JX, Kurth-Nelson Z, Kumaran D, Tirumala D, Soyer H, Leibo JZ, et al. Prefrontal cortex as a meta-reinforcement learning system. Nature Neuroscience. 2018;21:860–868. doi: 10.1038/s41593-018-0147-8 29760527

[17] Yang GR, Joglekar MR, Song HF, Newsome WT, Wang XJ. Task representations in neural networks trained to perform many cognitive tasks. Nature Neuroscience. 2019;22:297–306. doi: 10.1038/s41593-018-0310-2 30643294 PMC11549734

[18] Tsuda B, Tye KM, Siegelmann HT, Sejnowski TJ. A modeling framework for adaptive lifelong learning with transfer and savings through gating in the prefrontal cortex. Proceedings of the National Academy of Sciences. 2020;117(47):29872–29882. doi: 10.1073/pnas.2009591117 33154155 PMC7703668

[19] O’Reilly RC, Frank MJ. Making Working Memory Work: A Computational Model of Learning in the Prefrontal Cortex and Basal Ganglia. Neural Computation. 2006;18:283–328. doi: 10.1162/089976606775093909 16378516

[20] Kim R, Sejnowski TJ. Strong inhibitory signaling underlies stable temporal dynamics and working memory in spiking neural networks. Nature Neuroscience. 2020;24. 33288909 10.1038/s41593-020-00753-w

[21] Bonnen T, Yamins DLK, Wagner AD. When the ventral visual stream is not enough: A deep learning account of medial temporal lobe involvement in perception. Neuron. 2021;109:2755–2766. doi: 10.1016/j.neuron.2021.06.018 34265252 PMC10870832

[22] Yamins DLK, Hong H, Cadieu CF, Solomon EA, Seibert D, DiCarlo JJ. Performance-optimized hierarchical models predictneural responses in higher visual cortex. Proceedings of the National Academy of Sciences. 2014;111:8619–8624. doi: 10.1073/pnas.1403112111 24812127 PMC4060707

[23] Chung S, Abbott LF. Neural population geometry: An approach for understanding biological and artificial neural networks. Current Opinion in Neurobiology. 2021;70:137–144. doi: 10.1016/j.conb.2021.10.010 34801787 PMC10695674

[24] Sorscher B, Ganguli S, Sompolinsky H. Neural representational geometry underlies few-shot concept learning. Proceedings of the National Academy of Sciences. 2022;119(43):e2200800119. doi: 10.1073/pnas.2200800119 36251997 PMC9618072

[25] Tsuda B, Pate SC, Tye KM, Siegelmann HT, Sejnowski TJ. Neuromodulators generate multiple context-relevant behaviors in a recurrent neural network by shifting activity hypertubes. bioRxiv. 2022. doi: 10.1101/2021.05.31.446462 PMC13492707 41637720

[26] Gallego JA, Perich MG, Miller LE, Solla SA. Neural Manifolds for the Control of Movement. Neuron. 2017;94(5):978–984. doi: 10.1016/j.neuron.2017.05.025 28595054 PMC6122849

[27] Nieh EH, Schottdorf M, Freeman NW, Low RJ, Lewallen S, Koay SA, et al. Geometry of abstract learned knowledge in the hippocampus. Nature. 2021;595:80–84. doi: 10.1038/s41586-021-03652-7 34135512 PMC9549979

[28] Okazawa G, Hatch CE, Mancoo A, Machens CK, Kiani R. Representational geometry of perceptual decisions in the monkey parietal cortex. Cell. 2021;184(14):3748–3761.e18. doi: 10.1016/j.cell.2021.05.022 34171308 PMC8273140

[29] Marshall L, Bays PM. Obligatory encoding of task-irrelevant features depletes working memory resources. Journal of Vision. 2013;13:1–13. doi: 10.1167/13.2.21 23420420 PMC3587390

[30] Hochreiter S, Schmidhuber J. Long short-term memory. Neural Computation. 1997;9:1735–1780. doi: 10.1162/neco.1997.9.8.1735 9377276

[31] Sutton RS, Barto AG. Reinforcement Learning: an Introduction. 2nd ed. Cambridge, MA: MIT Press; 2018.

[32] Mnih V, Badia AP, Mirza M, Graves A, Harley T, Lillicrap TP, et al. Asynchronous Methods for Deep Reinforcement Learning. In: JMLR, editor. Proceedings of the 33rd International Conference on Machine Learning (ICML). vol. 48. New York; 2016. p. 1928–1937.

